# Lessons learned from scaling up a community-based health program in the Upper East Region of northern Ghana

**DOI:** 10.9745/GHSP-D-12-00012

**Published:** 2013-03-21

**Authors:** John Koku Awoonor-Williams, Elias Kavinah Sory, Frank K Nyonator, James F Phillips, Chen Wang, Margaret L Schmitt

**Affiliations:** a Ghana Health Service, Upper East Regional Health Administration, Bolgatanga, Ghana; b Ghana Health Service, Accra, Ghana; c Columbia University, Mailman School of Public Health, New York City, New York, USA

## Abstract

Ghana's Community-Based Health Planning and Service (CHPS) initiative is envisioned to be a national program to relocate primary health care services from subdistrict health centers to convenient community locations. The initiative was launched in 4 phases. First, it was piloted in 3 villages to develop appropriate strategies. Second, the approach was tested in a factorial trial, which showed that community-based care could reduce childhood mortality by half in only 3 years. Then, a replication experiment was launched to clarify appropriate activities for implementing the fourth and final phase—national scale up. This paper discusses CHPS progress in the Upper East Region (UER) of Ghana, where the pace of scale up has been much more rapid than in the other 9 regions of the country despite exceedingly challenging economic, ecological, and social circumstances. The UER employed 5 strategies that facilitated scale up: (1) nurse recruitment from their home districts to improve worker morale and cultural grounding, balanced with some social distance from the village community to ensure client confidentiality, particularly regarding family planning use; (2) prioritization of CHPS planning and continuous review in management meetings to make necessary modifications to the initiative's approach; (3) community engagement and advocacy to local politicians to mobilize resources for financing start-up costs; (4) a shared and consistent vision about CHPS among health administration leaders to ensure appropriate resources and commitment to the initiative; and (5) knowledge exchange visits between new and advanced CHPS implementers to facilitate learning and scale up within and between districts.

## Abstract (precis)

The original CHPS model deployed nurses to the community and engaged local leaders, reducing child mortality and fertility substantially. Key scaling-up lessons: (1) place nurses in home districts but not home villages, (2) adapt uniquely to each district, (3) mobilize local resources, (4) develop a shared project vision, and (5) conduct “exchanges” so that staff who are initiating operations can observe the model working in another setting, pilot the approach locally, and expand based on lessons learned.

## INTRODUCTION

Community-based health services programs are being launched and expanded throughout sub-Saharan Africa. Yet clinic-focused services remain the mainstay of most of these programs despite several convincing demonstrations that community-based operations can be more effective if static services are augmented with active provision of doorstep care.^1-4^ Even the most successful of these small-scale projects often fail to be mainstreamed into large-scale operations, because experimental trials generally use many resources that are challenging to replicate in large-scale operations.^5^ Moreover, when doorstep service innovations are expanded beyond original target areas, a complex process of instituting system-wide adjustments to new supervisory structures, leadership dynamics, policies, resource allocation strategies, and plans are needed at each organizational level.^6^ Such systemic changes are more complex to undertake than donors, researchers, and planners typically anticipate,^7^ largely because leadership required to develop and test small-scale innovations sometimes contrasts with the type of managerial and political leadership required to change a large-scale system.

Leadership to develop small-scale innovations is often different than the type of managerial and political leadership required to change systems at scale.

As early as the 1978 Alma Ata Conference, policies for achieving community-based primary health care became a pillar of Ghana's health policies. By the 1990s, however, mounting evidence that health development was not achieving national goals stimulated deliberations on feasible means of achieving health-sector reform.^8-13^ Moreover, the specific means of improving program performance remained unclear.^14-15^ Research identifying gaps in health outcomes called for national solutions, yet there was little concrete guidance to evidence-based policymaking and program development.

This paper describes the history of how an experimental study set the stage for a national program for promoting community-based primary health care—the Community-Based Health Planning and Services (CHPS) initiative. The paper also discusses factors of the initiative in the Upper East Region (UER) where CHPS was originally tested and scale up was most successful, despite being Ghana's poorest and most remote region. After a decade of implementation, the population of CHPS communities served by the CHPS program as a proportion of total district populations in the UER was 5 times the coverage achieved in the other 9 regions. To clarify factors that could explain the relative success of scale up, we consulted current and former Regional and District Directors of Health Services in the UER and compared their insights with those of leaders from an adjacent region where CHPS has been relatively slow to scale up.

## WHAT IS CHPS?

CHPS is a national health policy to reorient primary health care services from subdistrict health centers to convenient community locations. Its goal is to transform the dynamics of rural health care service delivery from community health care providers who passively wait for patients into outreach workers who actively seek patients in communities and their homes, also known as doorstep services. The vision of CHPS is to accelerate progress toward Millennium Development Goals 4 and 5 (MDG 4 and 5) on child health and maternal health, respectively. The core strategy entails deploying trained and salaried nurses, known as community health officers (CHOs), to village locations where they provide basic preventive, curative, and promotional health services in homes or community clinics. The CHOs are supported by the health care program's community organizational activities, including the recruitment, training, and deployment of volunteer workers. Critically important to CHPS is the effective provision of family planning information and services, to include doorstep provision of oral, injectable, and barrier contraception, referral for IUDs and other long-acting methods, and promotional services that are targeted to the needs of men and organized mainly by male volunteers.^16-17^

## PHASED PROGRAM DEVELOPMENT OF CHPS

**FIGURE 1.**
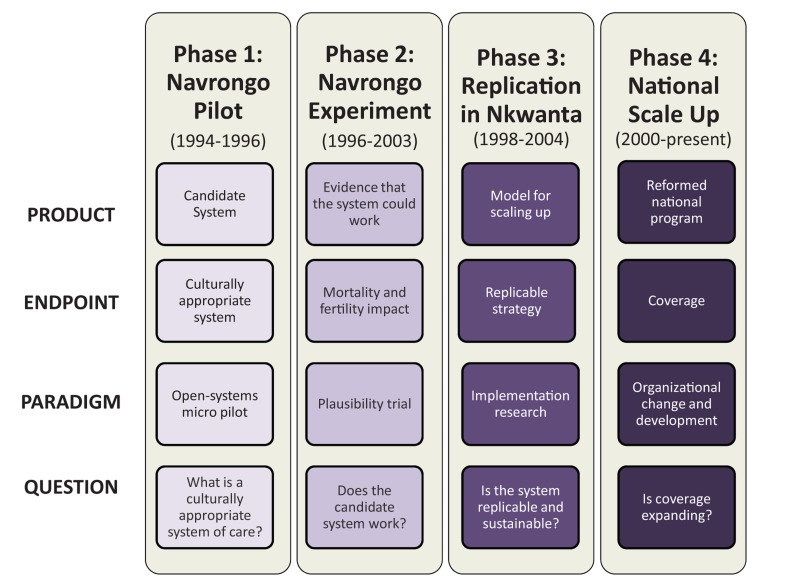
Phases in the Ghana Program Development Process Source: Reference 14.

Beginning in the early 1990s, Ghana instituted a partnership between applied health researchers and administrators to develop an action-oriented research agenda to guide health sector reform by resolving policy debates.^18^ CHPS was informed significantly by national program development experience in Asia.^19^ It was initially developed as a pilot project of the Navrongo Health Research Centre and progressed into a national policy over 4 overlapping phases (Figure 1).

### Phase 1: Navrongo Pilot to Develop Social Grounding for Service Strategies

In Navrongo, Kassena-Nankana District, UER, an 18-month pilot was initiated in 1994 by a team of social researchers, health scientists, and program implementers. The team consulted chiefs and elders, married women and men, and health care providers about appropriate strategies for implementing, managing, and sustaining community-engaged primary health care^14^ in order to subordinate operation of the program to social institutions that shape reproductive preferences and health-seeking behavior.^20-21^ Research scientists conducted qualitative research to determine the form of social interaction necessary for simplifying health communication and program mobilization processes and for facilitating volunteerism, consensus building, and ideational change. Qualitative research also identified gender issues and possible strategies for addressing them,^22^ including how to offset gate-keeping constraints in seeking health care,^23^ how to engage the support and participation of men in reproductive health promotional activities that they might otherwise resist,^24^ and how to sustain worker accountability for responsible service delivery.^17^ Focus group discussions were conducted quarterly to understand health worker and community reactions and to revise and tailor program operations. Following 18 months of this participatory research and planning, an appropriate model was finalized.^25^

Two sets of activities emerged from the Phase 1 model:

1. Existing clinical nurses were reoriented to community health care and assigned to village locations with the new designation of “community health officers” (CHOs). Nurses completed 18 months of training focused on basic curative health services, public health, immunization, and family planning.
2. Male volunteers from the communities were recruited and trained for 6 weeks to provide a limited set of services, such as oral rehydration and provision of condoms (Box 1). These *zurugelu* (“togetherness”) activities were based on existing traditional forms of governance, consensus building, and volunteerism, and they were designed to build male leadership and participation into reproductive health services, in addition to expanding women's participation in seeking reproductive and child health services. The project equipped volunteers with bicycles and start-up kits of essential drugs, conducted training on service management, and set up revolving accounts so that the community financed and sustained the flow of supplies.^26^

**Box 1. Community Health Services Piloted in Phase 1**

**Role of nurses,** known as community health officers (CHOs), who received 18 months of technical training and provided both health post-based and doorstep services:

- Integrated management of childhood illness (treatment of malaria and febrile acute respiratory infection with antibiotics), management of diarrheal disease, and referral of complicated cases
- Outreach organizational support for comprehensive childhood immunization
- Provision of micronutrient supplementation
- Antenatal care, including the provision of iron folate
- Support for uncomplicated deliveries and referral for emergencies
- First aid for minor injuries and skin conditions
- Health promotion and education, including supervisory support for volunteers
- Family planning counseling and services (oral contraception, condom distribution, and injectable contraception) and referral for long-acting methods
- Management of contraceptive side effects and referral, as needed

**Role of male volunteers,** who were selected by community stakeholders and trained for 6 weeks:

- Antipyretics for the care of children
- Oral rehydration
- Condoms
- Vitamin supplementation
- Health and family planning promotion directed mainly to male social networks
- Organizational backstopping of CHO activities, such as immunization and antenatal care

During Phase 1, the project clarified, documented, and translated operational details of mobilizing *zurugelu* and community health officer activities into practical and culturally appropriate implementation plans.^17^ See Box 2 for key lessons learned during the first phase.

**Box 2. Key Lessons from the Phase 1 Navrongo Pilot**

- Community-based consultation is an effective means of adapting strategies to the social environment. Traditional social structures can be successfully engaged as a programmatic governing body—in this case, the chieftaincy and lineage system.
- Collaboration between community leaders, implementers, and social scientists permitted “learning by doing”—adjustments of strategy according to new evidence, community advice, and worker comments. Operational aspects involving selection and training of workers and volunteers, as well as gender and communication strategies, work routines, location of health posts, and other operational details, can be optimized with “learning by doing” community input.
- Appropriate worker recruitment strategies need to be developed to address Ghana's cultural and language diversity. (Ghana has 82 languages.) National centralized recruitment and training of frontline workers lead to the deployment of staff who lack basic linguistic skills and cultural knowledge. District-level decentralized manpower development reduces turnover and improves performance.
- Workers should be posted to their home district but not to their home village. Villages seek nurses who have an element of social distance and an ability to maintain family planning confidentiality. For this reason, the project constructed “community health posts” to provide a balance between integrating services into the local context and maintaining social distance between the service and social system.

### Phase 2: Navrongo Experimental Trial to Test the Pilot Approach

**FIGURE 2.**
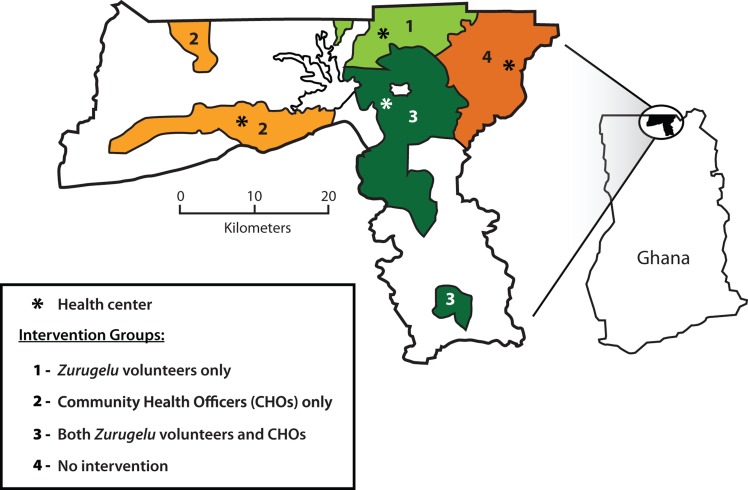
Navrongo Experimental Trial Intervention Groups, Kassena-Nankana District, Ghana Source: Reference 32.

Following the pilot, 37 communities of Kassena-Nankana District were grouped into experimental areas where *zurugelu* and CHO activities were deployed. Subdistricts were assigned to 1 of 4 experimental groups to evaluate the relative efficacy of the approaches (Figure 2):^16^

1. *Zurugelu* activities
2. CHO activities
3. Both *zurugelu* and CHO activities
4. No intervention

Relative efficacy was evaluated with data on child mortality and fertility trends collected by the Navrongo Demographic Surveillance System (NDSS).^16^^,^^27^^,^^28^

Results indicated that comprehensive community-based health care provided by the CHOs with support from *zurugelu* volunteers was associated with statistically significant reductions in fertility rates and substantial improvements in child survival compared with other experimental groups.^29-30^ In the first 3 years of the project, the total fertility rate declined by 1 birth in the combined CHO and *zurugelu* communities while it remained unchanged at nearly 5.5 in the comparison areas. In the communities where CHOs were deployed—whether with or without *zurugelu* volunteers—child mortality declined by one-half in only 3 years. Within 7 years, child mortality declined by two-thirds.

Deploying nurses, with support from community volunteers, substantially reduced both fertility and child mortality rates.

Communities in which CHOs were deployed but without support from community volunteers showed no changes in fertility rates. In addition, there was no fertility or mortality impact in the subdistricts where only *zurugelu* activities were implemented. In fact, volunteers posted without resident CHOs were less effective than providing no community care at all.^22^^,^^31^ The non-intervention group was also ineffective, experiencing modest demographic changes unrelated to community-based care.

Because the CHO plus *zurugelu* approach produced improvement in both reproductive and child health, the combined approach was deemed to be the optimal strategy for national scale up.^29-30^,^32-33^ See Box 3 for lessons learned during the second phase.

**Box 3. Key Lessons from the Phase 2 Navrongo Experimental Trial**

- The combination of traditional social institutions and volunteers with community health nurses, relocated from subdistrict health center clinics to communities, maximizes social acceptability of health and family planning care.
- The combined CHO and *zurugelu* volunteers approach produced substantial improvements in both fertility and child mortality rates.
- Piecemeal approaches do not work: Volunteer-based outreach without a resident CHO had no impact on fertility or child mortality; CHO-based services without the support of volunteers had no family planning and fertility impact.

### Phase 3. Replication Experiment in Other Districts to Validate Approaches

Although results of the Navrongo trial were impressive, district and regional health managers questioned relevance of the model to a national program. They debated whether replication of this model could be achieved in other parts of Ghana and whether it would improve demographic and health outcomes in areas outside the UER. Critics asserted that non-replicable institutional resources of the Navrongo Health Research Centre were responsible for the project's success, compromising relevance of the approach in other settings. Others argued that the Navrongo model was relevant only to the sociocultural circumstances of the UER.

To gain the necessary credibility for launching a national scale up of the model, the Navrongo experience was validated in another cultural and ecological zone of Ghana—the rural Nkwanta District of the Volta Region—whereby only routinely available resources of the Ghana Health Service (GHS) were used.^14-15^ To facilitate this process, the Volta Regional Health Administration sponsored a “knowledge exchange,” in which health workers from Nkwanta District visited the Navrongo project team in Kassena-Nankana to observe the Navrongo model of care and engage in candid discussions with counterparts about the feasibility of transferring the Navrongo model to Nkwanta. Although participants viewed the transfer of the model as requiring a daunting expansion of logistics capabilities, manpower, and supervisory workloads, direct dialogue with the Navrongo project team dispelled mystery about the CHPS development process and nurtured teamwork for developing pilot CHPS areas in Nkwanta.^34^

To facilitate implementation, the Nkwanta management team adopted Navrongo's participatory planning process for adapting strategies to local and contextual circumstances as several distinct differences existed between the Nkwanta and Kassena-Nankana Districts, including their social organizational structures. Moreover, Navrongo had extensive research resources that the Nkwanta team lacked. Thus, the Nkwanta team developed training, logistics, and management information procedures that could be implemented at minimal cost.^34-35^

The exchange program achieved operational success and contributed to increased health access and use.^35-36^ In response, the GHS sponsored additional replication projects so that each region of Ghana would have learning localities where CHPS could be implemented using the Nkwanta scaling-up strategy. In each replication district, the community-based care model increased contraceptive prevalence, participation in antenatal and postnatal care, and childhood immunization coverage.^34^^,^^36^

The success of the multidistrict replication process suggested that the Navrongo model could be implemented in similarly impoverished and health-deprived localities even with contrasting administrative, social, and cultural systems (Box 4). The exchange program also demonstrated that inter-district peer-exchange programs could be used to scale up CHPS.^40-41,14^ Most importantly, monitoring evidence suggested that participatory team exchanges were a more successful strategy for fostering scale up than workshops. Indeed, even today, most CHPS coverage is concentrated in districts that participated in exchanges with the Navrongo or Nkwanta teams. Much of the success with CHPS scale up in the UER is related to the fact that all workers in the region have had experience with the process of developing and implementing CHPS, even before they started the process in their home localities.^15^

Exchange visits between newly implementing and advanced teams were more successful at scaling up the CHPS model than traditional workshops.

**Box 4. Key Lessons from the Phase 3 Replication Trial**

- Knowledge exchange visits between new and experienced CHPS districts can catalyze the transfer of CHPS implementation capabilities.
- While CHO doorstep service delivery is the core component of CHPS, optimizing implementation requires adjustment to social and ecological conditions of each district. CHPS operational details should be locally planned and decentralized.
- Cultural heterogeneity requires strategic decentralization. Sustaining and spreading the Phase 1 pilot learning process informs and catalyzes scaling up.
- Redesigning training manuals to address technical inadequacy and improving documentation so that manuals are system implementation-focused rather than focused solely on health interventions was necessary.
- Decentralizing human resource development to the regional level ensured linguistic and cultural diversity were not constraints to scale up.

### Phase 4. National Expansion of the CHPS Initiative

**FIGURE 3.**
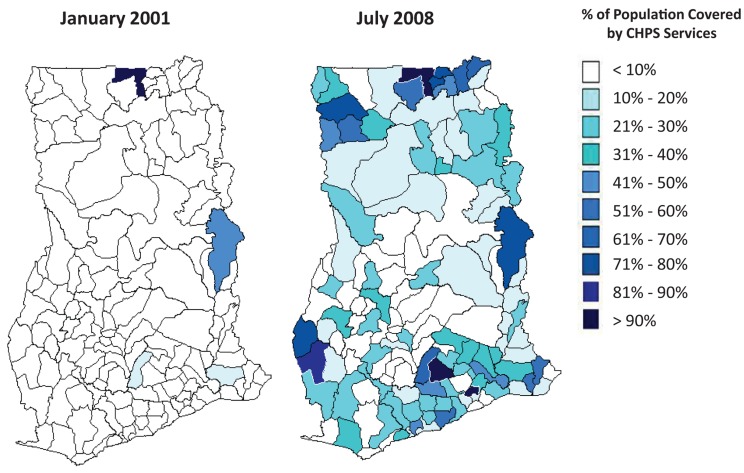
Geographic Density of CHPS Coverage by District, Ghana, January 2001 and July 2008 Abbreviations: CHPS, Community-Based Health Planning and Services. Source: Reference 15.

In 1999, the GHS reconvened district and regional health managers to assess the Nkwanta validation experience, and consensus emerged for promoting lessons from the Kassena-Nankana and Nkwanta Districts into a national program (Box 5). According to monitoring data from the GHS,^42^ nearly all districts in Ghana had some degree of coverage of the CHPS program by 2008 (Figure 3). Further observation and monitoring indicated that CHPS spread most rapidly in districts where pilots had been launched, suggesting that scale up followed patterns of change characteristic of diffusion processes.^15^^,^^43^

**Box 5. Key Lessons from Phase 4 National Scale Up**

Because core financing for CHPS is lacking, relying on other strategies helped to mobilize the necessary resources and accelerate scale up. These included:

- Funding “pilot CHPS” zones within districts that give managers experience while politicians can observe operations and witness community enthusiasm for CHPS. Let the political gains of CHPS expansion become a political gain for local assemblymen and assemblywomen.
- Seeking local government and community support to fund start-up costs while health sector managers seek financing from the development sector for expansion costs
- Prioritizing CHPS in regional staff meetings, budget discussions, and data review to catalyze organizational change. Follow through with regional technical staff visits to districts and CHPS zones, making CHPS expansion a key factor in performance reviews.

## CHPS SCALE-UP CHALLENGES AND UPPER EAST REGION SOLUTIONS

**FIGURE 4.**
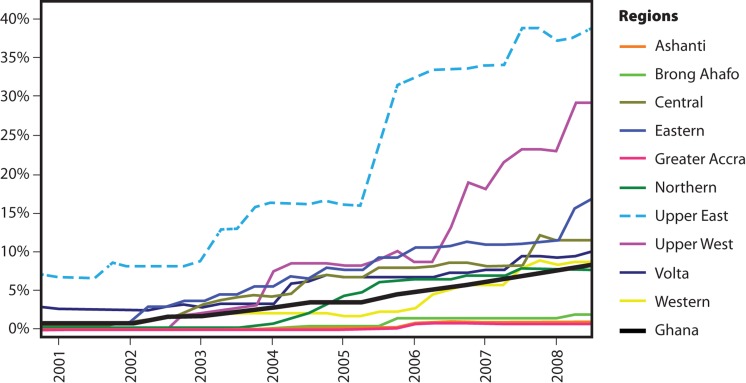
Percentage of the Population Served by Workers of the CHPS Program, by Region and Nationwide, September 2000 to June 2008 Abbreviations: CHPS, Community-Based Health Planning and Services. Source: Reference 15.

Monitoring data from September 2000 to June 2008 indicate that the proportion of the population covered by functioning CHPS zones across Ghana's 10 regions is low, with the most populous regions in southern Ghana performing the most poorly (Figure 4). The national trend of CHPS scale up in Ghana during this time period is also low (indicated by the black line in Figure 4). In contrast, the prevalence of CHPS coverage in the UER was 5 times that of the national average as of mid-2008. Further progress since 2008 has sustained the region as the leader among all 10 regions and the only region on target to attain full coverage by 2015.

### Methodology for Assessing Views of District Directors

To clarify stakeholders' perceptions of factors that either constrained or accelerated the scale up of CHPS in the UER, we interviewed all former and current District Directors of Health Services, 3 former and current Regional Directors of Health Services, and key staff and scientists of the Navrongo Community Health and Family Planning Project. For comparison, we interviewed the current Regional Director of the Northern Region and District Directors of Health Services in 4 northern regional health administrations. We also conducted a desk review of archival reports and documents of the GHS.

We identified 2 sets of challenges to scaling up the CHPS program.

In general, issues related to **manpower numbers, training, service capacity, and deployment** summarized in Table 1 apply to the early CHPS implementation era. For example, CHOs in the early phases were unprepared to deliver essential health services, such as addressing maternal and neonatal complications. In addition, the initial training program for CHOs did not provide them with the necessary skills in how to engage with the community. Since 2009, a project of the GHS known as the Ghana Essential Health Intervention Programme (GEHIP) has focused on diagnosing barriers to CHPS expansion and developing interventions to address these problems. For example, the project trained CHOs in strategies for saving newborn lives and implemented a referral system to manage complicated cases.

**Table 1.** CHPS Scaling-Up Constraints and Responses in the Upper East Region (UER) Related to Recruitment, Training, and Deployment of Community Health Officers

Constraint Type	Barriers to Scaling Up	Actions Implemented in the UER	Global Implications
Limited range of services	**Deficient range of services.** Community health officers (CHOs) were unprepared for essential services (midwifery, emergency management, immediate post-delivery care). **Over-extension of job descriptions**	Piloted and scaled up community-engaged referral system Trained CHOs in strategies for saving newborn lives Focus roles on the burden of disease and family planning	**Risk transition**. Community-based primary health care reduces the burden of disease. Emergency-related causes comprise an increased proportion of the remaining unaddressed burden**.** **Community-based planning**. Developing effective referral systems requires adapting operations to community road conditions and communication needs.
Inappropriate CHO recruitment	Insufficient nurse manpower Centralized recruitment results in deployment of workers to localities where they are not conversant with local languages or customs.	Expanded nurse training school volume Recruited trainees from districts where they are to be assigned and involved health committees in selection process	**Bottom-up planning.** Community health systems development requires “bottom up” strategic planning so that scale up builds capacity that effectively links services to local cultural conditions, languages, and health needs. **Plan for ethnic diversity**. Community-engaged recruitment reduces turnover and improves performance, morale, and community ownership.
Inappropriate CHO training	**Pre-service training.** Existing 18-month training program does not address community engagement, service outreach, and community health care planning. Overreliance on didactic training and shortage of locations and equipment for mentoring arrangements hinder CHO preparedness. **In-service training.** Relocating nurses from clinics to villages requires training them to be community organizers with liaison and diplomatic skills.	Implemented 6-month regional CHO internships focused on community engagement Organized peer mentoring coordinated with the training school curriculum	**Systems approach to manpower development.** Equipment and budgetary planning should integrate the process of pre-service, internship, and in-service training and plan for peer-mentoring arrangements. **Community-engaged peer leadership.** Didactic health technology training is insufficient.
Inappropriate CHO deployment	Insufficient programmatic focus on household services; health posts are the main service point. The National Health Insurance Scheme (NHIS) incentivized static services at the expense of doorstep care, reducing access. NHIS reimbursement for the provision of clinical services de-emphasizes supervisory outreach.	Developed supervisory work routines that are independent of NHIS reimbursement rules	**Systems approach to CHO deployment, monitoring, and supervision**. Programs that focus narrowly on a single community health worker cadre, health problem, or function are risky. “Learning localities” are needed where systems functioning is comprehensively monitored and where lessons learned are communicated to senior officials. **Compatibility of reimbursement schemes with doorstep care.** National Health Insurance schemes require careful trial of their impact on non-clinic based community-based service operations.
Inappropriate volunteer deployment	Volunteers providing antipyretics can inadvertently delay parental health-seeking behavior, elevating risk. With careful training and supervision, however, volunteers can provide integrated management of childhood illness (IMCI).	Training volunteers in social engagement methods is essential. Female health volunteers are more committed to service activities than male volunteers, but male volunteers are critical to family planning promotion.	**Risk mitigation with field research:** Reliance on untested imported initiatives is risky. **Partial IMCI does not work:** Volunteer services can cause more harm than good unless volunteer deployment is coordinated with deployment of trained nurses and governed by rigorous supervision.

Table 2 summarizes constraints related to **support systems for expanding CHPS, maintaining operations, and leading the program development process.** For instance, stakeholders pointed to the complicated health management information system that overburdened staff to the detriment of service delivery. Furthermore, lack of feedback on the collected data resulted in little use of the information to improve service delivery. In many districts, lack of leadership and political engagement, coupled with the absence of a budget line item for CHPS, resulted in inadequate resources and a lack of focus on and clarity about the program.

**Table 2.** CHPS Scaling-Up Constraints and Responses in the Upper East Region (UER) Related to Support of District Health Systems

Constraint Type	Barriers to Scaling Up	Actions Implemented in the UER	Global Implications
**Information Systems**			
Cumbersome information systems	Unwieldy Health Management Information Systems (HMIS) require more staff time for data management than is available for service delivery.	Simplified registers from 27 to 5 Developed monitoring tools for outreach and supervisory support	Inappropriate information systems can impede worker commitment to scaling up.
Lack of information utilization	Lack of feedback or systems for information utilization	Developed simple-to-implement data visualization tools	Implementation and supervisory support information is neglected in HMIS design.
Lack of essential information	Absence of actionable information about perinatal risks and causes of death	Developed maternal and neonatal mortality audit scheme with weekly medical review of results	Training and staff development require tools for evidence-based planning.
**Essential Equipment, Supplies, and Facilities**			
Shortage of community-based health facilities	High cost and slow pace of health post construction Official restrictions on the use of Ghana Health Service revenue for construction	Constructed interim facilities through community engagement and by volunteers Leveraged financing of construction through outreach to district political and development-sector leadership	Community investment in construction can facilitate engagement in health systems development.
Lack of essential equipment	Shortage of motorbikes and ambulances Lack of electrification, wells, and amenities	Obtained support from UNICEF and other donors for essential equipment, solar panels, and batteries	Low-cost equipment can be expensive to maintain. Investment in electrification and amenities reduces worker turnover and supports scale up.
Lack of essential commodities	Stockouts of essential drugs Expansion of services without expansion of access to supplies	Implemented simple stock monitoring and logistics reporting tool	Total systems planning is essential to effective community-based service development.
**Planning and Resources**			
Lack of financial planning and budgets	Absence of a budget line for CHPS	Implemented District Health Planning and Reporting Toolkit (2010)	Slow scale up can be addressed by clarifying resource management requirements and the health rationale for community-based services to grassroots politicians and leaders.
Lack of flexible resources	Extreme constraints on resources for the Common Fund Cash flow delays	Leveraged financing of the Common Fund (3 districts only)	CHPS lacks earmarked support from international donors. Instead, external resources are focused on technical assistance. Requiring a resource-constrained system to invest in incremental resources is unrealistic.
**Leadership and Governance**			
Lack of leadership for CHPS	Absence of district and regional leadership for CHPS implementation Lack of facilitative supervision	Implemented peer leadership exchanges between Navrongo and district teams and between leading district teams and counterparts Implemented supervisory peer leadership exchanges	Leadership is developed through transfer of knowledge via onsite demonstration and participatory exchanges. Workshops are an ineffective tool for leadership development.
Failure to replicate Navrongo community engagement	Lack of community entry and engagement Limited focus on establishing community health committees Absence of mechanisms for durbars and community exchanges	Employed social engagement strategies, including outreach to chiefs and elders, engagement with social networks and opinion leaders, community durbars for building consensus and collective action	Social engagement, gender strategies, and traditional governance strategies can be diluted with scale up. Resources for exchanges, demonstration, and discussion of social organizational issues can be crucial to effective scale up of community health service strategies.
Absence of political support	Absence of political engagement strategies Limited district development investment in health	Mobilized resources for health post construction through grassroots political support	Siloing community health development in the health sector can detract from scale up. Grassroots political engagement can contribute to offsetting resource limitations.

### 5 Strategies for Sustaining and Accelerating Scale Up in the UER

Interviews with the district and regional directors revealed 5 common strategies in the UER to cope with these challenges, which facilitated better scale up in the region compared with other regions.

#### Appropriate Manpower Recruitment, Training, and Deployment Strategies

Due to the close proximity of the the Navrongo Centre to the Regional Health Administration in the UER, which deploys CHOs, the operational details of launching, sustaining, and scaling up a large cadre of community nurses, including developing appropriate strategies for selecting trainees, has always been well understood by regional health leaders. Before 2002, the UER lacked a community health nursing school, and most nurses came from outside the region. They often did not speak local languages, and it was challenging to find nurses for community relocation who had the cultural understanding that community work required. As one district director noted:

It is all about commitment. When meet[ing with] them we plead with them because we know [it] is not easy traveling every day in and out … [and it] is risky. It's very difficult for us to draw [nurses] from [subdistrict] health centers. Some are pregnant, some are going to school, [and] some are joining their new husbands.

In response, the UER opened a regional training school in Navrongo and pioneered a policy of recruiting nurse trainees from the districts to which they would be posted. The communities themselves nominate and financially support most nurse candidates to ensure language competency. This early attention to “community-engaged” manpower development has catalyzed scale up. Unlike in other regions, where nurses are often unfamiliar with their place of assignment, CHOs graduating from the UER training school in Navrongo return home to work among their own community. In 2004, the year the first group of nurses graduated from the Navrongo training school, all districts in the region witnessed an increase in the number of functional CHPS.

Community involvement in nurse selection catalyzed scale up of the CHPS program in the Upper East Region.

#### Evidence-Based Review and Modification of CHPS Strategies

Results of the Navrongo research project were shared with the District Health Management Teams (DHMTs) from the onset of the experiment, which provided district managers with the evidence needed to convince them to make the necessary changes in operations. Furthermore, a continuous review of research in management meetings and a general climate of openness to the role of research in guiding action prompted additional modifications to improve the CHPS program. For example, long-term observation of the Navrongo project showed that neonatal mortality remained unacceptably high while health among other under-five children had improved dramatically.^29^^,^^44^ The CHPS approach, as it was originally developed, lacked sufficient focus on ways to prevent maternal and neonatal death and disability.^45^ To address this, UER regional and district health managers have invested in a program of in-service training designed to expand the range of services CHOs provide, with particular focus on improving and saving newborn lives, referral services, and other priority programs inadequately addressed by the initial Navrongo model.

#### Community Engagement and Grassroots Political Action to Mobilize Resources

The Navrongo project uncovered the need to develop health posts in which nurses could live and provide care to the community, which would establish a balance between community engagement and social distance. While the communities preferred nurses who were familiar with local languages and customs, they wanted the nurses to be socially removed from their own networks and extended families—someone the project termed as a “trusted outsider,” or a person who would keep secrets about family planning and avoid favoritism that might arise if the nurse was too closely linked to kindred groups.

To bypass GHS restrictions on spending resources for construction of new facilities, the UER Regional Health Administration encouraged communities to develop interim facilities with local labor, donated materials, and traditional construction methods. Then managers approached district assemblymen and development officers to replace functioning interim facilities with more permanent structures. A decade of systematic problem solving with community engagement has been the single most important factor explaining the rapid scale up of CHPS in the UER.

Community engagement has been the single most important factor in scaling up the CHPS program in the Upper East Region.

#### Shared and Consistent Vision and Commitment Among Leaders

Regional and district leaders in the UER have had a priority focus on and commitment to CHPS. From the onset, the UER Health Administration carried out strategies to mobilize resources, even cutting budgets of other activities and temporarily curtailing services to create budgetary space for CHPS. As UER district health managers noted when they were asked about scaling-up problems:

*Resources are not always as you need. There are always other things that you can put in there. If there is not that commitment to implement CHPS, you don't go that extra mile to put resources in place*.

The response [to shortage of funding] is we just keep going and going. Because you cannot ignore them. You keep trying; sometimes you get lucky, in another time you are not.

If we get to the [district] assembly and explain things to them, [they] understand.

We discuss [CHPS] with the NGOs. We would draw our budget and discuss our [funding] challenge with them [and clarify] that this is what we want to do. But this is our challenge—to throw more light on the program so that they would help us.

Although launching CHPS is not expensive, its incremental costs are difficult to sustain. The estimated total expenditure for 1 fully functional CHPS zone covering a population of about 3,500 is US$33,345 (about US$9.50/capita) (including a solar panel but excluding CHO salaries, fuel for vehicles for monitoring activities, and training costs). The most costly components are facility construction ($20,240) and motorbike procurement ($5,300). However, if village volunteers conduct construction, using traditional material for building walls, the cost is reduced substantially (to about $3/capita). These modest costs are nonetheless a major challenge for district managers, who often lack adequate resources for implementing even the most basic health service agenda. The incremental costs of initiating the Navrongo model was the most dominant constraint to CHPS implementation identified during the course of our interviews. Every district director interviewed noted some aspect of this problem. For example:

There is no funding for CHPS. The strategy was mainly to cut back on other things. It's tough.

For me, funding is a big issue. Even when you got District Assemblies to build a compound [health post], really getting it functional, getting people trained, and getting all the logistics is the problem.

Where you want a new system to work well, you put money for it. CHPS doesn't have [money].

When I want to [send nurses] to school I have to look for my own funding. No one funds training. The CHOs were first supported by the district assemblies but they say they are constrained, so they are now funding themselves.

Everything they [District Assemblies] do depends on their Common Fund. If the Common Fund doesn't come, whatever they tell you, it is always difficult because the internally generated revenue is not adequate to even meet their overhead courses.

CHPS expansion has been most successful in areas where GHS regional and district officials have persuaded local governments to add CHPS into their annual budgets. Simple-to-implement communication strategies have facilitated exchanges between local government authorities, politicians, and health leadership about CHPS. For example, GHS leaders have involved local officials in CHPS-sponsored community celebrations of implementation progress, linking popular support for CHPS to the political aspirations of elected officials.

CHPS expansion was most successful in areas where local politicians contributed funds to the program's budget.

But not all district health directors—the local focal points of health administration—consider CHPS as the most effective means to achieving health objectives, partly because most managers are unfamiliar with the Navrongo experiment but also because many are unconvinced that CHPS is cost-effective. To address these issues, the UER is testing a toolkit that will help managers compare different budgetary options and their implications on offsetting health and mortality risks. This tool has provided district managers with practical means of demonstrating the potential impact of investing in CHPS on health outcomes and may have contributed to accelerating the expansion of investment in CHPS between 2010 and 2012.^42^

In addition to their commitment to CHPS, UER leaders also shared a coherent and consistent vision about CHPS, which contributed to the scaling-up process. According to GHS policy documents, CHPS is defined as “the mobilization of community leadership, decision-making systems and resources in a defined catchment area (termed a ‘zone’), the placement of reoriented frontline health staff, known as Community Health Officers, with logistics support and community volunteer systems to provide services according to the principles of primary health care (PHC-Plus).” Regional and district leaders in the UER had a similar interpretation of this policy. For example, most UER directors considered CHPS to be functional even without a compound as long as CHOs provided services. In contrast, directors based elsewhere tended to disagree with this perspective. One such manager noted:

You need a person to be there. You need a compound [health post] to make it functional. Those are the two key ingredients you need … CHPS without a compound compromises [the program]. At any time, people should be able to call on you … People have been trying to define that it's functional without a compound. But a compound must be there. In a community, when services are not done holistically 24 hours, how can you say it's functional?

Some issues are not clear[ly] defined. What is operational CHPS? What is functional CHPS? [We] Need to make sure that [the] document is still relevant; [we] need to redefine to clarify parameters. CHPS is progressing at various stages. It needs revision. People have different understandings. People have expressed different views. Based on that, the policy should be revised.

Similarly, officers interviewed in the UER were consistently clear about responsibilities of the CHOs, but CHO responsibilities were less clearly defined elsewhere. For example, one manager outside the UER argued that CHPS workers should be health promoters but not service providers:

[We] Shouldn't overload them [the CHOs]. CHOs should not be doing antenatal [care] and immunization. They should only do education … [and] should only be going out. [They] Should not be sitting there.

The shared vision and commitment to the CHPS program among UER leaders facilitated the process of political and community engagement, which in turn facilitated resource mobilization. When health sector leadership catalyzes the process of community engagement and resource mobilization, CHPS scale up proceeds even if no health sector revenue exists for the program.

#### Peer Exchange Visits

Exchange visits between new and advanced districts helped to facilitate scale up by giving new CHPS program leaders an opportunity to directly observe how the model was working successfully. Dialogue between program leaders dispelled mystery about the CHPS development process and nurtured teamwork among the implementers. Peer exchanges were designed to achieve this by equipping visiting teams with the capability to implement the program in 1 or 2 zones.

To focus exchanges on implementation, visiting teams were encouraged to include participants who could represent the contrasting implementation responsibilities of each level of the system: the District Health Management Team, at least 1 subdistrict supervisory team, and 2 or more CHOs from the participating subdistrict. These individuals were then teamed with counterparts from an advanced implementation team to plan together how to implement a functioning CHPS zone in the participating team's home district.

The teams discussed the practical task of zone implementation, community engagement, replicating exchanges between communities, and building local political commitment to CHPS expansion. Each participating team also received seed funds to cover the cost of implementing a pilot zone, with training on how to cost and budget the program. Taken as a set of activities, exchanges equipped and financed pilot implementation of CHPS in ways that catalyzed scale up of operations once participants returned to their home districts.

Participants were also oriented to focus group methods that would enable managers to respond to community and worker opinion, adapt strategies to local conditions, decentralize planning, and take ownership of the program as a district-directed, scaling-up initiative.

Although 38 districts throughout Ghana participated in exchanges, this process was far more intense in the UER than elsewhere. By 2004, all districts, subdistricts, and CHOs in the region had participated in 1 or more exchanges with the Navrongo team. National monitoring data have since revealed that peer-exchange visits have been more successful than workshops. In fact, by 2008 CHPS coverage was concentrated in the 38 districts that participated in exchange visits with the Navrongo or Nkwanta advanced CHPS districts.^15^

## CONCLUSION

Despite the challenges that have been identified, the CHPS initiative has begun to introduce health care reform in every region and district of Ghana. In the UER, where scale-up problems have been the focus of strategic review, trial, and dissemination, CHPS has established a sustainable service model, which ensures that progress is adapted to local realities and guided by evidence. Coverage of community-based care in the UER was 5 times the national average after a decade of scaling up, even though the region is the poorest in Ghana.

Nonetheless, problems with district health system leadership persist and merit further research, demonstration, and policy development. The new Ghana Essential Health Intervention Programme (GEHIP) has been launched to address the evidence gap on how to improve district leadership for continued CHPS expansion,^39^ including the development of district planning tools, resource management systems, and intersectoral coordination strategies.^46^ In addition, the new project is testing a simplified management information system to meet the information needs of CHOs and district leaders while minimizing the time and effort required for CHOs to capture, manage, and report information.^47^

Although much remains to be accomplished, the CHPS experience in the UER attests to the practicality of scaling up CHPS throughout Ghana. Its core strategy is based on the principle of putting evidence into action through research, experimentation, multiple validations, and adaptation efforts—learning by doing.
